# Supplementary figures and images for: Genome-wide selection inference at short tandem repeats
Source: PLoS Genet. 2025 Dec 1;21(12):e1011959. doi: 10.1371/journal.pgen.1011959 (PMC12680348; doi:10.1371/journal.pgen.1011959)

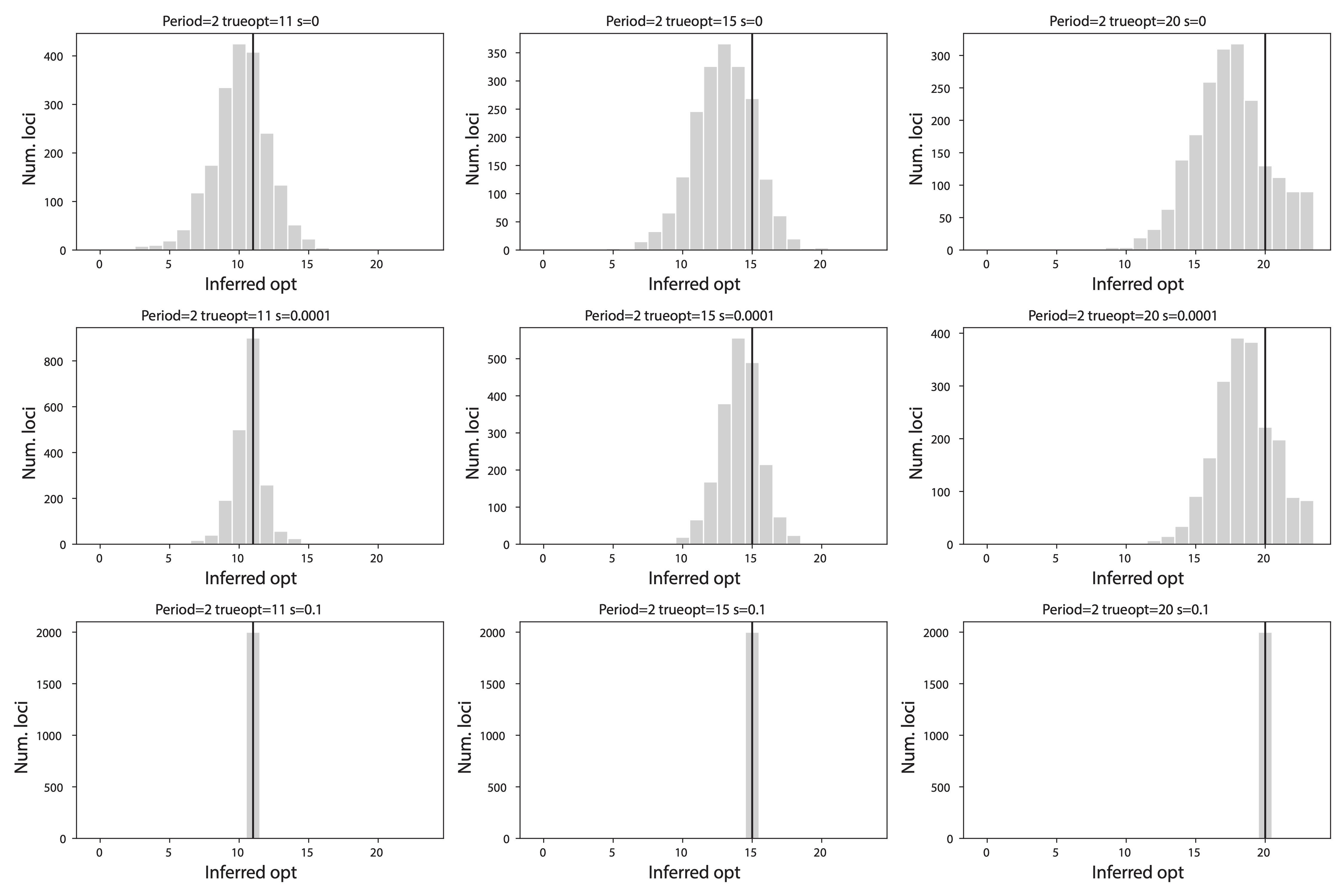

Supplement: S2 Fig — For each setting tested, we performed forward simulations of 2,000 STR loci using the procedure described in Methods. In each panel, the x-axis gives the inferred optimum (modal allele) and the y-axis gives the number of simulations. Gray histograms show the distribution of the inferred optimum (modal allele). Black vertical lines show the simulated optimum allele. Top row: s = 0, middle row: s = 0.0001; bottom row: s = 0.1. Left, middle, and right panels show simulations for dinucleotides with true optimum alleles of 11, 15, and 20, respectively. (TIF) [file pgen.1011959.s008.tif]

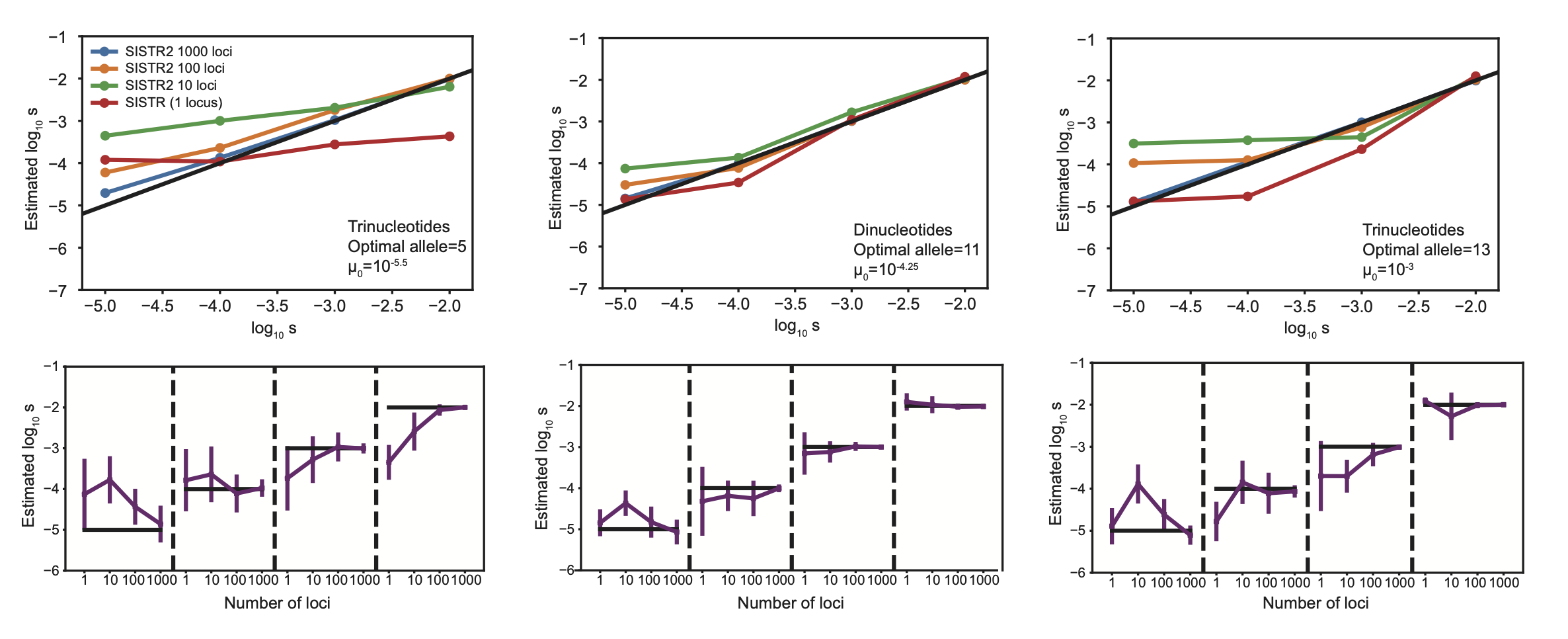

Supplement: S6 Fig — We tested SISTR2 under various conditions (left = short trinucleotides with low mutation rates, middle = short dinucleotides with moderate mutation rates, right = long trinucleotides with high mutation rates). For each condition, we evaluated SISTR2’s selection inferences as a function of the number of STR loci used as input. In the top panels, the x-axis gives the simulated mean value of s and the y-axis gives the inferred mean value across 20 estimates. Colors denote the number of loci used for inference (red = 1, green = 10, orange = 100, blue = 1,000). Bottom plots show the mean + /- 1 s.d (purple) across the 20 simulations in each condition. True mean values of s are given by black horizontal lines. For all simulations shown, gamma distribution parameter a (i.e., the shape parameter) was set to 0.6. (TIF) [file pgen.1011959.s012.tif]

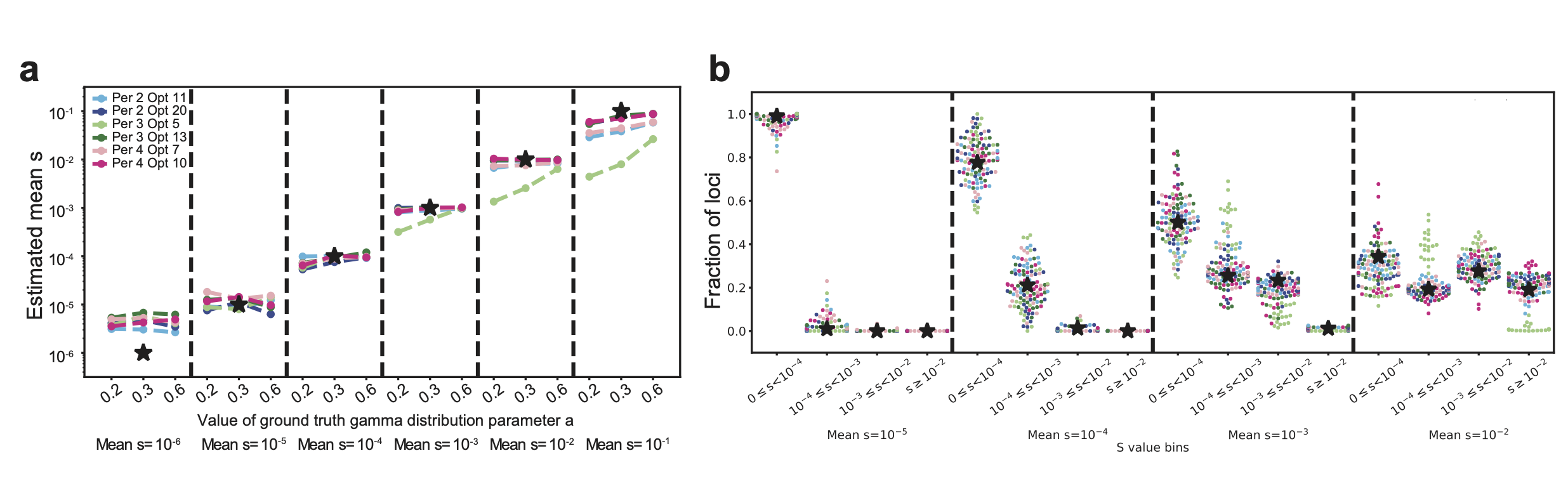

Supplement: S7 Fig — (a) and (b) are the same as Fig 1b-c, except “observed” allele frequencies contained simulated STR genotyping errors (Methods). Our results demonstrate that except in cases with extremely low underlying mutation rates (e.g., short trinucleotides), modest genotyping errors do not bias selection inferences. Short dinuc = optimal allele 11, long dinuc = optimal allele 20, short trinuc = optimal allele 5, long trinuc = optimal allele 13, short tetranuc = optimal allele 7, long tetranuc = optimal allele 10. (TIF) [file pgen.1011959.s013.tif]

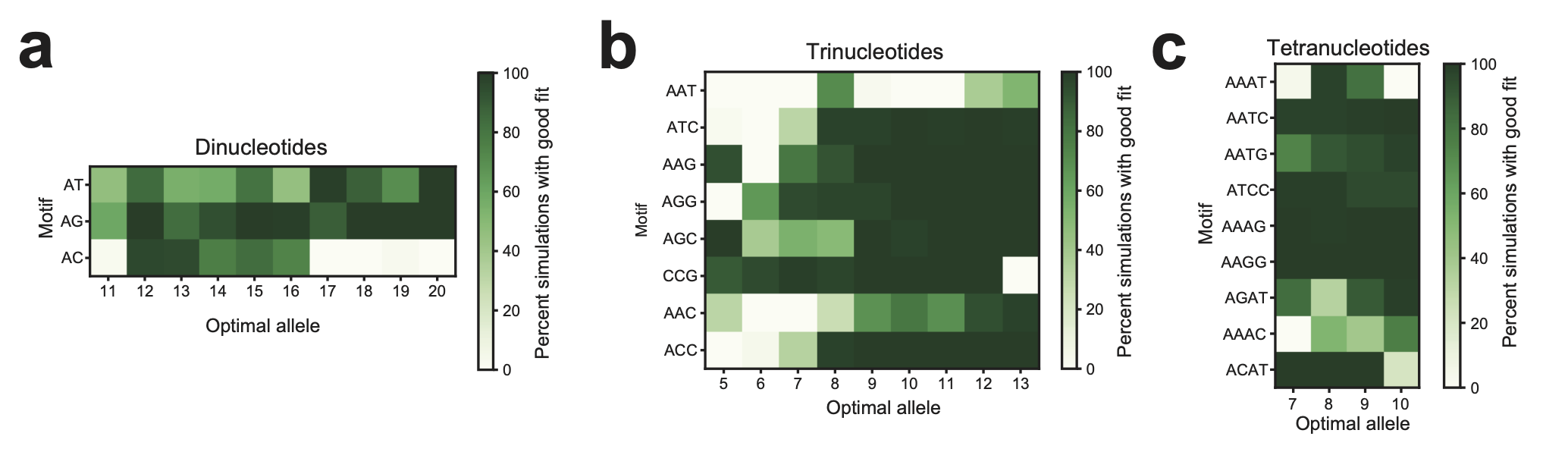

Supplement: S9 Fig — For each STR class, corresponding to a particular repeat unit (x-axis) and optimal allele (y-axis), we determined the mutation model with the best fit (Fig 2; Methods) and assessed the goodness of fit to observed data. For each class, we simulated 100 datasets based on the inferred mutation model, and compared the distribution of heterozygosities for simulated loci to those from observed loci using a KS test. Each heatmap cell shows the percent of simulation rounds with KS test p-value>0.05, indicating the two distributions are similar and the model fits well. Left = dinucleotides, middle = trinucleotides, right = tetranucleotides. (TIF) [file pgen.1011959.s015.tif]

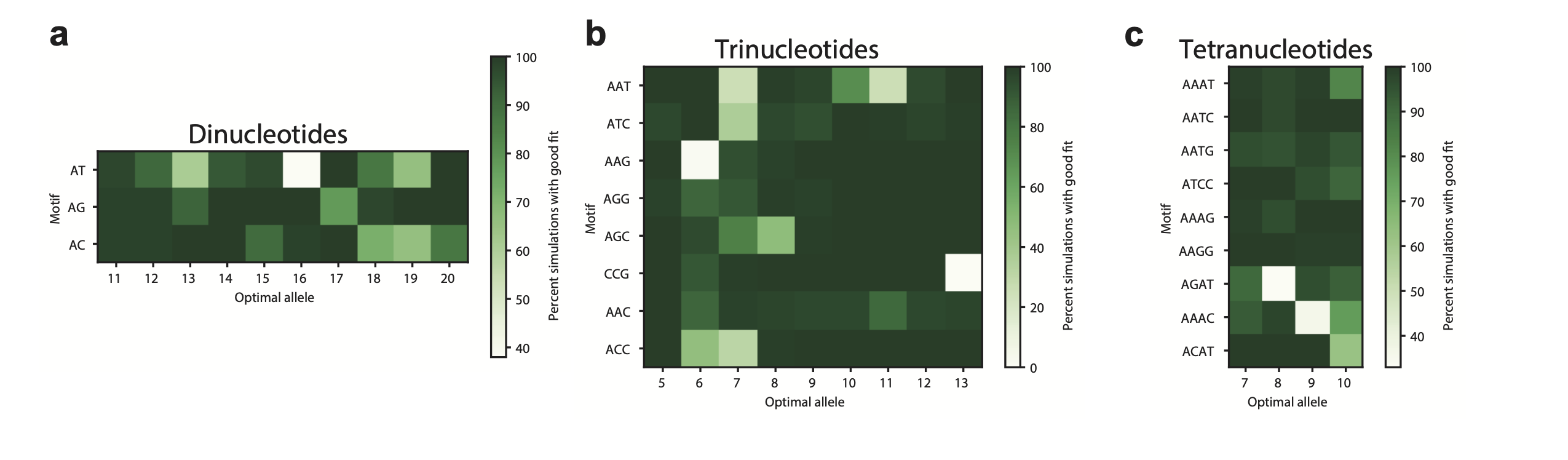

Supplement: S10 Fig — To reduce differences in power across different STR classes to detect differences between observed vs. simulated heterozygosity distributions, we repeated the goodness of fit analysis (S9 Fig) but including at most 50 STRs sampled from each class. Panels are the same as in S9 Fig. Left = dinucleotides, middle = trinucleotides, right = tetranucleotides. (TIF) [file pgen.1011959.s016.tif]

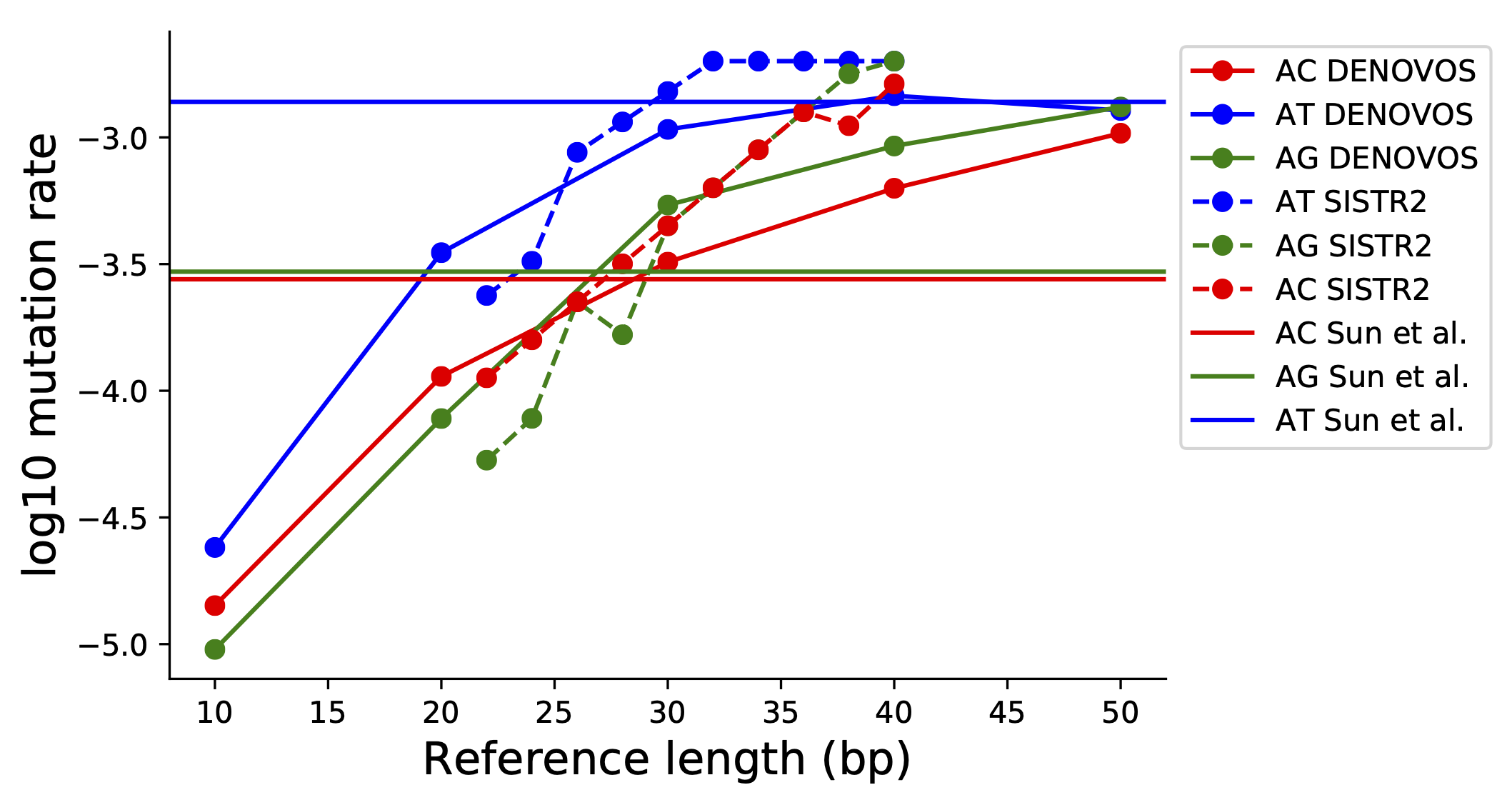

Supplement: S12 Fig — We compared the dinucleotide mutation rates inferred by SISTR2 (dashed lines) to those inferred from de novo mutations based on WGS of the Simons Simplex Collection (SCC) dataset [8] (solid lines with points) and from de novo mutations based on capillary electrophoresis from Icelandic individuals [16] (solid horizontal lines). The SISTR2 inferred mutation rate for each motif/optimal allele combination was set as the median mutation rate in the posterior of accepted mutation models from the feasible mutation parameters analysis (Fig 2). In all datasets, AT repeats have higher mutation rates than AC or AG repeats. Additionally, inferences from SISTR2 and in SSC show AC repeats mutate faster than AG repeats for shorter but not longer repeat tracts. (TIF) [file pgen.1011959.s018.tif]

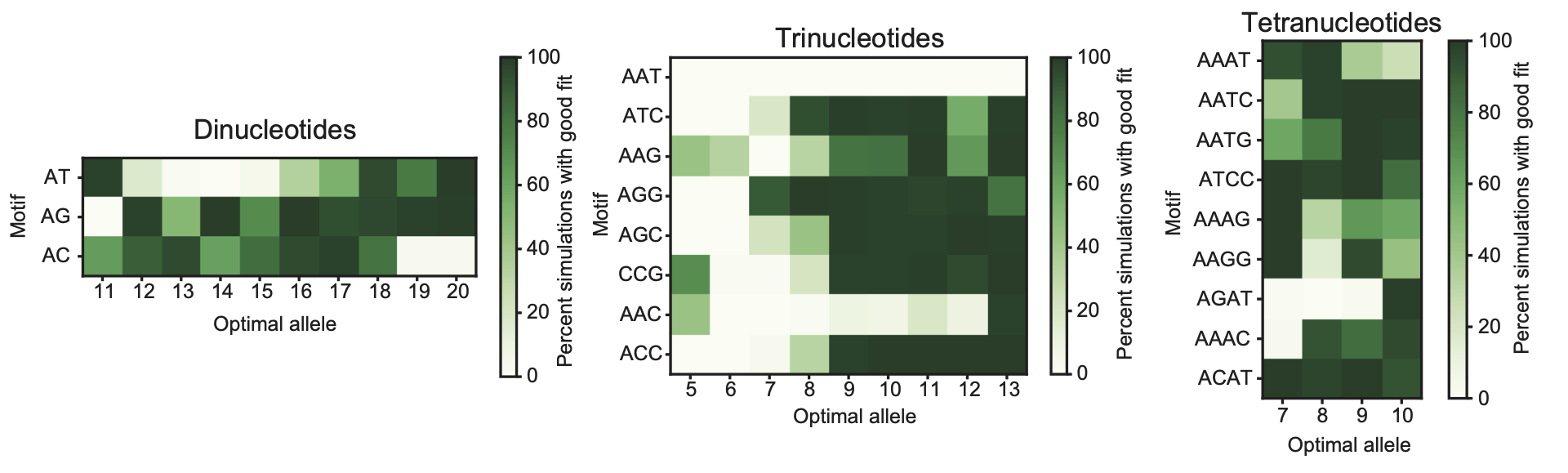

Supplement: S13 Fig — For each class of loci (i.e., optimal allele/repeat unit pair), 100 heterozygosity distributions were simulated using the distribution of selection coefficients inferred by SISTR2. Then, a KS test was used to compare the simulated distributions to the observed distributions. Each heatmap cell shows the percent of simulation rounds with KS test p-value>0.05, indicating the two distributions are similar and the model fits well. These values are reported as the KS Score in S2 Table. Left = dinucleotides, middle = trinucleotides, right = tetranucleotides. (TIF) [file pgen.1011959.s019.tif]
