# Supplementary material for: Genome-wide selection inference at short tandem repeats
Source: PLoS Genet. 2025 Dec 1;21(12):e1011959. doi: 10.1371/journal.pgen.1011959 (PMC12680348; doi:10.1371/journal.pgen.1011959)
Supplement: Attachment — Submitted filename: SISTR2_Revision_ReviewResponse-submit101525.pdf [file pgen.1011959.s024.pdf]

Review Response - PGENETICS-D-25-00338

Overview

We thank the reviewers for their careful reading of our manuscript and their helpful comments. We were encouraged by the positive feedback, and implemented the suggestions in our current manuscript version. We highlight here the major areas addressed in our revision.

1. **Assessing the robustness of SISTR2 to model misspecification.** Our initial manuscript contained analyses showing the robustness to genotyping errors and different numbers of input loci. We have expanded this to:
    a. *Assess robustness to optimal allele inference.* Our method makes the assumption that the modal allele is the optimum at each locus. We have provided additional analyses to interrogate how often this assumption holds (**Supplementary Figs. 1-2**). Overall, we found that as long as selection is at least modest, the modal allele is in most cases equal to or centered around the optimum (**Supplementary Figs. 1-3**). An exception is loci with relatively long optima under weak selection, for which the mode is frequently lower than the optimum (**Supplementary Figs. 2**), a trend driven by length-dependent mutation rates **(Supplementary Fig. 3**). We find that SISTR2 inferences are robust to expected minor differences in inferred vs. true optimum alleles (**Supplementary Fig. 8**). As expected, when the inferred optimum (mode) is very far from the true optimum (e.g. 11 vs. 20), which occurs very infrequently based on our simulations, SISTR2 inferences are biased (**Supplementary Fig. 8**).
    b. *Assess robustness to mutation model misspecification*. As expected, when the specified neutral mutation model does not match the model used to generate input allele frequencies, SISTR2 results are biased (**Supplementary Fig. 8**). This motivates our careful analysis to first identify accurate mutation models for real data (**Fig. 2**) before continuing with selection inference (**Fig. 3**).
    c. *Assess robustness to prior.* The only priors input to SISTR2 are on the gamma distribution parameters a and b. We found that our default prior performs well in all scenarios tested. We evaluated the impact of changes to this prior and found that it had a minor impact on SISTR2 inferences (**Supplementary Fig. 8**).
2. **Providing additional details for the SISTR2 ABC method.** We have substantially expanded the Methods section to more fully describe the mutation, selection, and demographic models as well as details regarding the forward simulation procedure. We additionally have provided more detailed rationale behind the mutation model used based on literature of empirical STR mutations. In the course of this revision, we now clarify that the selection coefficient *s* for SISTR represents the mean decrease in fitnesses across both alleles in the genotype. As such, 0.5*s* represents the heterozygous effect of an allele, which is most appropriate to compare to values of *s* for SNVs. Thus, for analyses comparing STRs to SNVs, we now present both the original SISTR value of *s*, as well as the scaled values (divided by 2). Though our comparisons between SNVs

and STRs remain qualitatively unchanged, we have edited the main text based on these scaled numbers and to describe the rationale for these comparisons.

Responses to additional comments: In-depth responses to each comment from reviewers are below. Reviewer comments are provided in blue and our responses are in black. Text revisions are denoted using Track Changes in the provided .docx file.

Critiques and response to Reviewer #1

Huang et al present SISTR2, a computational method that is designed to infer the distribution of selection coefficients for a set of short tandem repeats (STRs) based on their allele frequencies. STRs are implicated in some heritable diseases and have long been of interest in population genetics studies, but most methods for inferring selection focus on single nucleotide variants. I found this to be a nicely written and (overall) clear manuscript that makes a novel contribution to the population genetics literature. I do have a few comments and questions for the authors.

We thank the reviewer for the positive feedback and helpful comments, which we address below.

Main comments:

1. The overall logic of the method makes sense -- given an optimal allele and a set of STRs, selection will constrain the distribution of heterozygosities relative to a mutation-only model. As the authors note, inferring a mutation rate model is hence a critical step in the inference. Is it not also true that a model in which mutation rates vary across loci could explain some of the variation in heterozygosities across loci? It was not clear to me if/how mutation rate variation across loci (within a single class of STRs defined by the repeat unit) was considered in the study. As I understood it, a single best-fitting mutation rate model was fit for each class of loci (based on sequence content and repeat length), and the selection inference was performed on the basis of this mutation model. Is there a biological reason to think that mutation rate might vary across loci, and if so how would this affect the selection inference?

It is correct that our analyses use a single best-fitting mutation rate model for each class of loci, which is a simplification. It is certainly the case that mutation rates and other mutation parameters can vary across loci. However, the major sources of variation across loci are repeat unit length (and sequence) and the total length of the repeat (PMIDs: 22922873, 33442040, 27126583) (particularly the longest stretch of perfect repeat sequence). Both of these features are accounted for by fitting separate models for different STR classes (e.g. repeat unit/optimal allele length pairs). In our original development of SISTR2, we had experimented with drawing mutation parameters from a prior distribution for each class, rather than using static mutation models. These experiments had minimal impact on inference results.

There are other sources of mutation rate variation within classes that are not accounted for here. For example, mutation rates are more modestly influenced by features such as the

sequence composition of flanking regions (PMID: 37872149) or epigenomic features (PMID: 28892063). Another factor not accounted for here is sequence imperfections. Although our analysis here is restricted to “perfect” repeats with no imperfections in the reference genome, it is possible some loci have point mutations, such as SNPs or small indels that interrupt the repeat sequence, that could influence mutation rates (PMID: 11102705). To address this point, we have added a discussion of these limitations to the **Discussion.**

2. Description of ABC method: The approach uses ABC (approximate Bayesian computation) to infer the distribution of selection coefficients. I think this is a great approach and a nice contribution. While I was able to follow the logic of the ABC method in broad strokes, several questions remained for me after reading through the methods. I will highlight my main questions here, but my general request is that the authors revise this section to improve the reproducibility.

We agree that in the original submission several details of the ABC method could have been described more clearly. We have substantially expanded the **Methods** section titled **SISTR2 mutation, selection, and demographic models** to provide more detailed descriptions of our models, as well as the rationale behind choices such as the summary statistics used.

2a. The method uses observed heterozygosity across a set of STRs as the summary statistic of interest in ABC. There are other statistics computed from the frequency distributions that could have been considered. Why was this statistic chosen? Were others considered, and is there any reason to think that this should be among the most informative possible choices?

We had considered multiple choices of summary statistics to capture variation at each STR, based on the allele frequencies at each one. (An important note is that whereas SNPs and other variant types often only have two common alleles, and therefore can be fully described by a single statistic, e.g. minor allele frequency, STRs are often multi-allelic and thus require a different choice of summary statistic.)

We had considered the frequency of the most common allele, or the number of common alleles at each STR as alternatives. However, these metrics only capture information about a subset of alleles. On the other hand, heterozygosity, defined as 1-sum_i p_i^2, where p_i is the frequency of the ith allele, summarizes the entire allele length distribution at a locus. Based on our initial evaluations, we found that using heterozygosity as a summary statistic gave the best results. We have added this rationale to the **Methods** section.

2b. The text states that the method "Compare(s) the distribution of the simulated heterozygosity for all loci within the set to the distribution of the observed heterozygosity for all loci in the set by sorting both vectors, obtaining the difference vector, and taking the mean of the absolute value of the differences." It sounds like this difference vector consists of the per-locus heterozygosities for each locus? If so, in my opinion this choice needs a little more justification. It seems like a few outlier loci in either the observed or simulated data could have a disproportionately large impact on the deviation between model and observed. Most ABC approaches summarize the

observed/modeled data with a few informative statistics to reduce the dimensionality of the difference calculation.

We have revised the text in Methods to clarify how we summarize the observed vs. modeled data: We first obtain vectors of heterozygosity values for each input STR and for the same number of simulated STRs (default to 1,000 loci or all loci if less than 1,000 loci are used as input). We then sort each of these two vectors of 1,000 heterozygosity values (one of the observed, one for the simulated STRs). Finally, we take the difference of these two vectors and summarize by taking the mean of the absolute value of the differences.

It is true that this mean absolute difference metric could in theory be impacted by outlier loci. Outliers from the simulated data would not be accepted by the ABC algorithm and we expect would not have a major impact, since we ultimately focus on the top 1% of simulated distributions with most similarity to the observed data. Extreme outliers in the observed data are more of a concern. However, in practice we found that for most classes of STRs we found good model fits, and inspection of heterozygosity distributions at classes of loci that could not be fit well (**Supplementary Fig. 11**) suggested that the poor fit was not driven by outlier loci. We now mention in the text that this approach could be sensitive to extreme outliers in the input data.

2c. The method assumes that the modal allele is the fittest allele, and in the Discussion it is said that " must analyze STRs with different optimal alleles separately". If I understand correctly, the authors pool together STRs with the same repeat unit (e.g., "AAAT" repeats), and in each round of selection inference include only alleles with a given observed modal allele. Is this correct? Or do they combine all repeats with a given repeat unit, and assume that a single fittest allele based on the mode across all loci in the set?

To clarify: SISTR2 is run separately on each set of STRs, where sets are defined by the repeat unit and modal allele. e.g. all AAAT repeats for which the model allele observed in the population is 7 copies would be considered together, all AAAT repeats with a modal allele of 8 would be considered together, etc. We have now clarified this both in the **Results** and **Methods**.

3. Validation of ABC

3a. If it is true that only loci with the same modal allele are used in the inference step, how does this assumption play into validation? When selection is strong, I would expect that the modal allele is typically the fittest. When selection is weak, I would suppose that the modal allele is not always the fittest allele. Have the authors considered how this might affect inference?

As noted above it is true that loci with the same modal allele are considered together in the inference step. To evaluate how often the modal allele has the highest fitness, we performed simulations under various mutation and selection parameters and determined how often the modal allele matches the optimal (added now as **Supplementary Fig. 1-2**). As expected, under at least modest selection ($s>\sim10^{-3.5}$), the modal allele is almost always the same as the optimum. On the other hand, as suggested by the reviewer, under weak selection (and high mutation rate), this assumption is often broken. In most cases under at least weak selection, the

distribution of the modal allele is centered around the true optimum (added now as **Supplementary Fig. 2**). An exception is loci with relatively long optima under weak selection, for which the mode is frequently lower than the optimum (**Supplementary Figs. 2**), a trend driven by length-dependent mutation rates **(Supplementary Fig. 3**). We find that SISTR2 inferences are robust to expected minor differences in inferred vs. true optimum alleles . As expected, when the inferred optimum (mode) is very far from the true optimum (e.g. 11 vs. 20), which occurs very infrequently based on our simulations, SISTR2 inferences are biased. This new analysis is shown in **Supplementary Fig. 8.**

3b. In general, the manuscript would benefit from some consideration of model misspecification. I don't think it is necessary to perform a sweep across many sets of parameters, but checking how assumptions about mutation models/symmetry, demography, etc in a couple of basic scenarios would be useful for assessing the robustness of the method.

We have now provided additional robustness analyses, described in the **Overview** section above, in **Supplementary Fig. 8** (in addition to **Supplementary Figs. 1-2** exploring the assumption that the modal = optimum allele).

4. Empirical literature on STR mutation rates
The manuscript does a nice job of summarizing other population genetics studies of mutation rates and selection on STRs and putting this study into context, but there is also quite a lot of molecular genetics research on STRs. I'm not an expert on this literature, so I'm not going to suggest specific papers, but in general the paper would benefit from more linking back to the empirical literature on STRs. What is known (or at least previously studied) for STR mutation rates from pedigrees or other studies? How do these mutation rate estimates compare to those in this study?

We have substantially expanded the section of the **Methods** now titled **"SISTR2 mutation, selection, and demographic models"** where we describe the mutation model used to refer back to the extensive literature on STR mutations that have motivated the model choices used here. Mutation rates inferred in our study show strong concordance with rates obtained from trio-based analysis of de novo mutations (see **Supplementary Fig. 12**) which is noted in the text.

Minor comments:

It's not entirely clear to me why we can assume that the empirical STR loci (genotyped with GangSTR and used to infer mutation models) evolve neutrally — this could be clarified.

We focused intergenic STRs as a set of presumably most neutral loci for this analysis. Although a few examples of pathogenic intergenic STR mutations are known, we expect the vast majority of intergenic repeats are under at most weak selection. We have now noted this in the text.

I was confused by the statement "The model with the highest number of ABC acceptances corresponds to the maximum likelihood mutation model" (and other references to maximum likelihood). Maximum likelihood is a different statistical approach from ABC which depends on computing a likelihood function, and in general model comparison is one of the things that is not straightforward to do with ABC. I think it's fine to allow the model with the most acceptances to be the "best-fitting" for the purposes here, but I don't think it should be called a maximum likelihood fit unless I have misunderstood the approach.

We thank the reviewer for pointing out the lack of clarity in our original manuscript. We intended to convey that under a uniform prior, parameter values will be accepted in proportion to their likelihood. As such, the proportion of acceptances would give us a Monte Carlo estimate of the likelihood of the parameters. However, we agree that our description was confusing. Throughout the manuscript, we now use the term “best-fitting” instead of “maximum likelihood” to describe the mutation models. Lastly, we have added a section to the methods titled “***Estimating feasible mutation rates***” in which we provide additional context on the estimation of mutation models. In total, we believe that our manuscript more clearly describes our inference of the mutation model parameters.

The Discussion notes that the mutation model is symmetric — this was not clear to me earlier in the manuscript and (unless I missed it) this information should be stated earlier.

We apologize that this was a typo - it meant to say the selection (not mutation) model is symmetric and in future work we will consider alternatives to this model. We have fixed this in the Discussion. We have additionally clarified in the results section **Overview and validation of the SISTR2 joint inference method** that the selection model is symmetric.

The discussion states "Further, it is not immediately clear why different STR loci with the same repeat unit would have different optimal lengths." I don't think it's too hard to think of models where this isn't true, such as a model where the selective effect is only due to STR length and the optimal length is dependent on local sequence context and not the specific base composition of the STR? If there is other literature supporting this assertion a citation would be helpful, otherwise I think the opposite point could also be claimed just as easily.

We agree with the reviewer that there are likely a variety of plausible explanations for why different STRs might have different optimal alleles. Since this could be argued either way and to avoid confusion, we have removed this sentence from the Discussion. We still state that extending our model to relax this assumption is a future goal.

Critiques and response to Reviewer #2

The authors present a new ABC method to jointly infer the distribution of fitness effects and mutational model of a set of STR mutations. The authors first show that their method correctly estimates parameters from the distribution of fitness effects via a set of simulations. Then, the authors show how well the method can infer the mutational model of a set of presumed neutral

STR mutations. Then, the authors apply their method to estimate the distribution of fitness effects of STRs with different classifications that depend on their functional annotation due to being on a particular region ("coding", "intron", "promoter", etc.) and also due to their particular motif. Finally, the authors interestingly show that the mutational burden of STR variants is bigger than the mutational burden of SNPs.

I think that the authors did a great job explaining their method and that the conclusions are very relevant to understand the functional importance of STR variation. I have a few major comments on the manuscript that are mostly focused on the assumptions behind the method and a few minor comments.

We thank the reviewer for their careful reading and helpful comments below.

Major comments

"Our mutation model is based on a generalized stepwise mutation (GSM) model with two modifications, including a length-dependent mutation rate and a directional bias in mutation sizes toward an optimal (central) allele length"

Is there a reason behind the selection of that model? If there is, it would be good to provide more information in the text. Can the authors provide some discussion on how the prior distribution of the mutational model chosen has an impact on their conclusions?

Based on comments from both reviewers, we have substantially expanded the description of the mutation model. We have also explained the rationale for our choices of model and parameters, which are based extensively on empirical data of STR mutations, in the **Methods** section **SISTR2 mutation, selection, and demographic models.**

Accurate mutation models and parameters are critical for our selection inference. For example, if we incorrectly assume a high mutation rate at a set of slowly mutating loci, we would tend to overestimate selection. Conversely, if mutation rate is set too low we could incorrectly infer that a set of loci that are under selection are neutrally evolving. We note that mutation rates inferred here, which are based on inference from standing variation in the population, show strong concordance with rates inferred across different STR types directly from de novo mutation (**Supplementary Fig. 12**). Further, our goodness of fit analysis of best-fit mutation models inferred for different repeat classes (**Supplementary Figs. 9-10**) demonstrates that the mutation models used here fit well to observed STR variation data. Taken together, these results suggest the mutation models used for selection inference are reasonable. Finally, in our added robustness analysis in **Supplementary Fig. 8**, we show as expected that misspecifying the mutation model can bias SISTR2 results which motivates our careful examination of feasible mutation models in **Fig. 2** before proceeding to selection inference on real data in **Fig. 3**.

Can the authors provide some guidelines or discussion regarding how to choose an appropriate prior distribution for all the parameters in the analyses presented?

SISTR2 models selection coefficients as being drawn from a gamma distribution, $\Gamma(a, b)$. The only prior distributions used are those on the parameters a and b (other parameters including mutation model parameters are set to fixed values based on empirical data and analyses described in the new **Methods** section "**Estimating feasible mutation rates**".

As described in the **Methods**, a is drawn from a uniform distribution from 0 to 1 and a*b (mean value of the distribution) is drawn from a log normal distribution with a mean and standard deviation of the underlying normal distribution log(0.0003) and log(30), respectively. The rationale for this choice is based on estimates of the DFE for deleterious SNVs. Previous work found that a leptokurtic (shape<1) gamma distribution fit well for both nonsynonymous and noncoding SNVs. Our prior on a*b is centered at 0.0003 and includes estimates of the mean *s* for noncoding (0.00066) and nonsynonymous (0.0065) mutations. We have updated the section of the text where the priors are described to mention this rationale. We additionally evaluate how making at least small changes to these prior parameters influences SISTR2 results (**Supplementary FIg. 8**).

"Indeed, recent work has suggested that the modal allele is the most influential summary statistic at inferring the optimal allele and in cases where it was possible to infer to infer the optimal allele, the optimal allele was typically the modal allele (in single optima models) or a multiple of the optimal allele (in periodic optima models)6."

I think this is a reasonable assumption but surely this depends on the fitness difference between the optimal STR allele and the other alleles. If the difference in fitness between the optimal allele and all the other alleles, in terms of Ns values, is larger than 1 then likely the modal allele will be the optimal allele. However, if the difference, in terms of Ns values, is smaller than 1 then likely the modal allele won't always be the optimal allele.
Could the authors provide more information that backs up the presented assumption? One possibility could be to do simulations under different Ns values and show that the modal allele is the optimal allele under different differences of Ns values between the optimal allele and all the other alleles. I am also open to other possibilities such as discussing more information from references that back up this assumption.

We thank both reviewers for helpful comments on this assumption, which encouraged us to provided a more detailed evaluation of how this influences our results. We described in the **Overview** the new analyses related to this in **Supplementary Figs. 1-2, 8**.

Indeed, as demonstrated in **Supplementary Fig. 2**, there are two main cases where the modal will not be equal to the optimum. One is the case of weak selection, in which case the mode is expected to be the optimum but shows a distribution centered on the true optimum. Another case we had not initially considered is at longer loci under up to modest selection. Because our model uses a length-dependent mutation rate, in which shorter alleles mutate more slowly than long alleles, loci with long optima tend to show bi-modal allele length distributions, and/or be biased toward alleles that are shorter than the optimum. This is because once a mutation occurs

that results in a shorter allele, due to the slower mutation rate those shorter alleles tend to accumulate (see **Supplementary Figs. 2-3**). We now expand on this point in the **Results** and **Discussion**.

Minor comments

Figure 1a) The caption does not describe what the blue variables mean.

We have updated the caption to describe the blue variables (these are parameters that are estimated by SISTR2). We have additionally expanded the caption to define all variables and edited the Methods to ensure all variables mentioned in **Figure 1** are defined. (We also updated **Supplementary Fig. 4** to use matching nomenclature).

Figure 2.- I would recommend adding more information in the caption about what the models a-f mean.

We have updated the **Figure 2** caption to add more details about how the different models are defined.

I am also unsure about why the Log10 mutation rate varies as a function of the repeat length. Is the repeat length an average of the repeat length across many STRs? Can the authors provide more information behind this behavior?

We have clarified in the caption of **Figure 2** that mutation rates are allele-specific. That is, at the same locus, different alleles have different mutation rates that depend linearly on their total length. This is consistent with the widespread observation from de novo mutations that mutation rates are strongly correlated with repeat length (see response to main comment #1 from Reviewer 1 above).

"For tetranucleotides, AAGG, AAAG, and AGAT, which also showed higher mutation rates than other tetranucleotide STRs13 (Fig. 2f),"

This is hard to see quickly in the figure. Can more information be provided that backs up this statement?

We have updated **Fig. 2d-f** to annotate on the plots that models a-f or a-g go generally from higher to lower overall mutation rates. We have also clarified in the legends that the models there correspond to those shown in **Fig. 2a-c**.

It would be good to show the compared "Total burden" on Table S5 and Table S6 to compare the total burden between SNPs and STR variants.

Thank you for this suggestion. We have added a "Total" (Nonsynonymous + CADD>15) burden to Table S6 and renamed the column in Table S5 previously named "Additive fitness" to "Total additive burden" to highlight the total burdens that are compared for SNPs and STRs in the main text.

“ is mutation rate of each allele x is determined as is the length-dependent mutation rate, such that the (μ) = (μ0) + ; β is the length constraint, indicating the bias of long alleles to contract and short alleles to expand toward the optimal allele length; ρ is the step size parameter describing the geometric distribution from which mutation step sizes are drawn.”
Can you explain in mathematical terms how β and ρ work?

We have now added mathematical details on how β and ρ work to the **Methods** section titled **SISTR2 mutation, selection, and demographic models.**

I might have missed that in the text but how do you define the “presumed neutral loci”?

We have now clarified in the text that we use intergenic STRs as presumed neutral loci.

Additional questions

Have all data underlying the figures and results presented in the manuscript been provided? Large-scale datasets should be made available via a public repository as described in the PLOS Genetics data availability policy, and numerical data that underlies graphs or summary statistics should be provided in spreadsheet form as supporting information.

Reviewer #1: No: Links to the summary statistics used in the study would be more helpful than only listing the SRA accession.

We have uploaded summary statistics used for this study to figshare: https://doi.org/10.6084/m9.figshare.30261118.

Reviewer #2: Yes
